# Treatment of Patients With HCV Related Cirrhosis: Many Rewards With Very Few Risks

**DOI:** 10.5812/hepatmon.6095

**Published:** 2012-06-30

**Authors:** Roberta D’Ambrosio, Alessio Aghemo

**Affiliations:** 1 First Division of Gastroenterology, Cà Granda Hospital Foundation IRCCS Maggiore Policlinico, University of Milan, Milan, Italy

**Keywords:** Hepatitis C, Liver Cirrhosis, Virology

## Abstract

Antiviral treatment of chronic hepatitis C virus (HCV) is aimed at the persistent eradication of the virus, the so-called sustained virological response (SVR), with the aim ultimately being to prevent the development of liver-related complications and improve patients’ survival. Patients with HCV-related compensated cirrhosis are the group most likely to benefit from viral clearance, as several retrospective studies have shown liver complications rates to be positively modified by the achievement of a SVR. Whether these benefits rely on viral clearance or on the histological improvements seen following successful interferon (IFn)-based therapies has recently been a matter for debate, as studies have shown cirrhosis to regress in some patients with a SVR. Whatever the mechanisms, cirrhosis has the uncanny ability to be both a dominant indication for therapy, as well as one of the strongest baseline factors associated with reduced efficacy of any IFn-based regimen. This has led to the development of alternative treatment strategies, such as low dose pegylated IFn (PegIFn) monotherapy, that unfortunately has proven to be of limited efficacy. For this reason regimens able to clear the virus without relying on the broad antiviral effect of IFN are eagerly awaited.

## 1. Background

Antiviral treatment of chronic hepatitis C virus (HCV) is aimed at persistent eradication of the virus, the so-called sustained virological response (SVR). However, the ultimate aim is to prevent the development of liver-related complications and improve patients’ survival. Such hard endpoints are difficult to achieve and demonstrate in patients with mild to moderate fibrosis stages, as liver-related complications in these patients occur infrequently and the main causes of death are to be found in causes not related to the liver [1][2]. In contrast, patients with HCV related compensated cirrhosis have an annual incidence of hepatocellular carcinoma, liver decompensation and esophageal variceal bleeding ranging from between 1 and 3% [3][4][5], that ultimately accounts for an annual mortality rate for liver related complications of between 2.7% and 6.7% [6]. Several retrospective studies [6][7][8][9][10] have shown such figures to be positively modified by the achievement of a SVR, effectively making patients with HCV compensated cirrhosis a high priority group to receive anti-HCV treatments. However, enthusiasm for treating patients with cirrhosis is somewhat limited by the still disappointing SVR rates that are achieved in this group of patients by interferon (IFN)-based regimens, as well as by the risk of developing serious treatment related adverse events (AEs), which are especially worrisome in some categories of patients such as those with a decompensated disease [11][12]. Still, HCV eradication in patients with compensated cirrhosis should remain a top hepatology priority, as it responds to the May 2010 resolution of the World Health organization (WHO), [13] that not only declares hepatitis to be an urgent global health issue, but also calls for the treatment of those most at risk of developing liver related complications.

## 2. The Impact of a SVR on the Natural History of HCV-Related Cirrhosis

Patients with cirrhosis due to HCV are at risk of liver-related morbidity and mortality [5][6] (Table 1), with antiviral treatment representing the only current option to modify the course of the disease. Although pivotal studies assessing the benefit of a SVR on HCV cirrhotics showed no benefits of viral eradication in patients with HCV-related advanced fibrosis [14][15], further studies have provided definitive results supporting a positive role of a SVR in terms of clinical events, by reporting reduced rates of liver complications among this subgroup of patients [7][8][9][10][11][16][17][18][19]. Therefore, the achievement of a SVR in cirrhotic patients with a HCV infection should be considered as a primary goal, when balancing the pros and cons of an antiviral treatment for these patients. With all the caveats related to the retrospective design, the relatively small sample size and marked heterogeneous population (different stage of disease, duration of follow-up, type of IFNα and schedule treatment) that limit their applicability, all the studies support a role for a SVR in reducing the incidence of liver decompensation (i.e. ascites, hepatic encephalopathy and gastrointestinal bleeding), the development of hepatocellular carcinoma (HCC) and liver-related deaths. Among the first to demonstrate the beneficial impacts of HCV eradication on the natural history of patients with HCV cirrhosis were Yoshida et al. [20], who retrospectively analyzed data from 2,890 patients (337 cirrhotics) with any degree of liver fibrosis, and they reported a reduced risk of HCC among cirrhotics with a SVR (RR = 4.78; 95% CI 1.13-20.18) when compared to those who failed the antiviral treatment (RR = 12.3; 95% CI 6.81). A subsequent retrospective analysis [7] demonstrated that the SVR cirrhotics had reduced rates of liver-related deaths, even if this result did not reach statistical significance (treated vs. untreated SVR 1/53 vs. non-SVR 15/177, 2% vs. 8%, P = 0.13). Similarly, a prospective, non-randomized, controlled study from Japan [15] reported the beneficial impact of a SVR on 271 cirrhotic patients treated with IFN and followed-up for 7 years after treatment completion, since patients with a SVR showed reduced rates of both HCC (11/64 vs. 73/207, 17% vs. 35%, P = 0.008) and liver-related deaths (0/64 vs. 32/207, 0 vs. 15%, P = 0.0002).

**Table 1:** Mean Weighted Annual Percentage Rates of Clinical events in Patients With Cirrhosis. Some part of data are derived from the study by Alazawi et al. [6]

	**Weighted Mean, %**
Complications	6.37
HCC	3.36
Ascites	2.69
Variceal bleeding	0.58
Encephalopathy	0.45
Jaundice	1.48
Death/transplantation	4.58
Liver failure	1.16
Varices	0.22
HCC	2.70
Sepsis	0.41
Non-liver	0.70

In Italy, 920 patients with compensated cirrhosis [9] who received IFN monotherapy were followed-up for a median period of 96 months after treatment completion; patients who achieved a SVR showed significant benefits in terms of liver-related complication reduction (0 vs. 1.88 per 100 person-years), HCC (0.66 vs. 2.10 per 100 person-years) and liver-related death (0.19 vs. 1.44 per 100 person-year) (P < 0.001). Finally, failure to achieve a SVR was associated with a higher risk of liver-related complications, HCC (RR 3.12; 95% CI, 1.42-6.86) and liver-related mortality (Hazard Ratio (HR) 7.59; 95% CI, 1.84-31.29). A more recent French study [11], designed to evaluate the relationship between the regression of cirrhosis and clinical outcomes in patients with HCV cirrhosis treated with IFN-based regimens , similarly reported that SVR patients were less likely to die of liver-related causes (3/39 vs. 19/57, 8% vs. 33%, P = 0.003) and displayed lower rates of liver-related complications (4/39 vs. 23/57, 10% vs. 40%, 0.001), that included HCC (3/39 vs. 14/57, 8% vs. 25%, 0.05), ascites (0 vs. 10/57, 0 vs. 18%, P = 0.005), and EPS (0 vs. 7/57, 0 vs. 12%, P = 0.04) development. Finally, two studies [10][19] analyzed the role of a SVR on the clinical course of patients with advanced liver fibrosis, including not only patients with cirrhosis, but also those with lower stages of fibrosis. Veldt et al. [10] retrospectively analyzed data from 479 patients staged S4 to S6 according to the Ishak scoring system [21], and they confirmed that a SVR was associated with a reduced risk of any liver-related event (4/142 vs. 87/337, 3% vs. 26%, P < 0.0001; adjusted HR 0.21; 95% CI 0.07-0.58, P = 0.003), including liver failure (0/142 vs. 42/337, 0 vs. 12%, P < 0.0001; HR 0.03; 95% CI 0.00-0.91). However, although patients with a SVR showed a reduction in HCC (3/142 vs. 32/337, 2% vs. 9%, P = 0.003) and liver-related deaths (1/142 vs. 16/337, 2% vs. 5%, P = 0.03) these differences were not statistically different (adjusted HR 0.46; 95% CI 0.12-1.70, P = 0.25 and adjusted HR 0.19; 95% CI 0.02-1.44, P = 0.107). In the study conducted by Cardoso et al [19], which included 307 patients with bridging fibrosis or cirrhosis (F3 and F4 by METAVIR) [22] followed-up for 3.5 years following the end of their treatment, incidence rates per 100 person-years of liver-related complications, liver-related deaths, and HCC were significantly lower in SVR than in non-SVR patients (0.62 vs. 4.16, 0.61 vs. 3.76 and 1.24 vs. 5.85, respectively; P < 0.001 for all comparison). Reviewing all of these studies, it is evident that the achievement of a SVR may reduce the risk of cirrhosis-related complications. However, viral eradication does not eliminate the risk of HCC, since liver cancer has been reported to occur even years after treatment completion, at a rate of between 0.66 and 1.24 per 100 person years [9][19] or between 0.6% and 2.5% per year [10][17][23][24]. Therefore international guidelines recommend that cirrhotic patients with a SVR should be kept under ultrasound surveillance, with the aim of obtaining an early HCC diagnosis [25].

on the contrary, no univocal recommendations have been stated with regard to endoscopic surveillance, since the data published so far are not conclusive. Two Italian studies [18][26] have prospectively investigated the role of a SVR on the course of portal hypertension, by using repeated esophagogastroduodenoscopies in patients who achieved a virological response, after treatment completion. Bruno et al. [18] followed 218 patients for up to 18 years, and found a SVR was able to prevent the development of esophageal varices (EV) (0% for SVR vs. 39.1% for non-SVR, P < 0.0001). On the other hand, another Italian study [26], in which 127 patients were followed for up to 108 months after the end of IFN-based regimens, showed EV development both in SVR and non-SVR patients, although the incidence of de novo EV were reduced among patients with a SVR (2/57 vs. 8/53, 3.5% vs. 15.1%, P = 0.047). Interestingly, despite the small number of patients with pre-treatment EV at the EGDS baseline, a progression in the EV size was demonstrated, independently of the treatment outcome (SVR 1/5 vs. non-SVR 2/12, P = 0.87).

## 3. Impact of a SVR on Liver Fibrosis and Cirrhosis Regression

**Figure 1:**
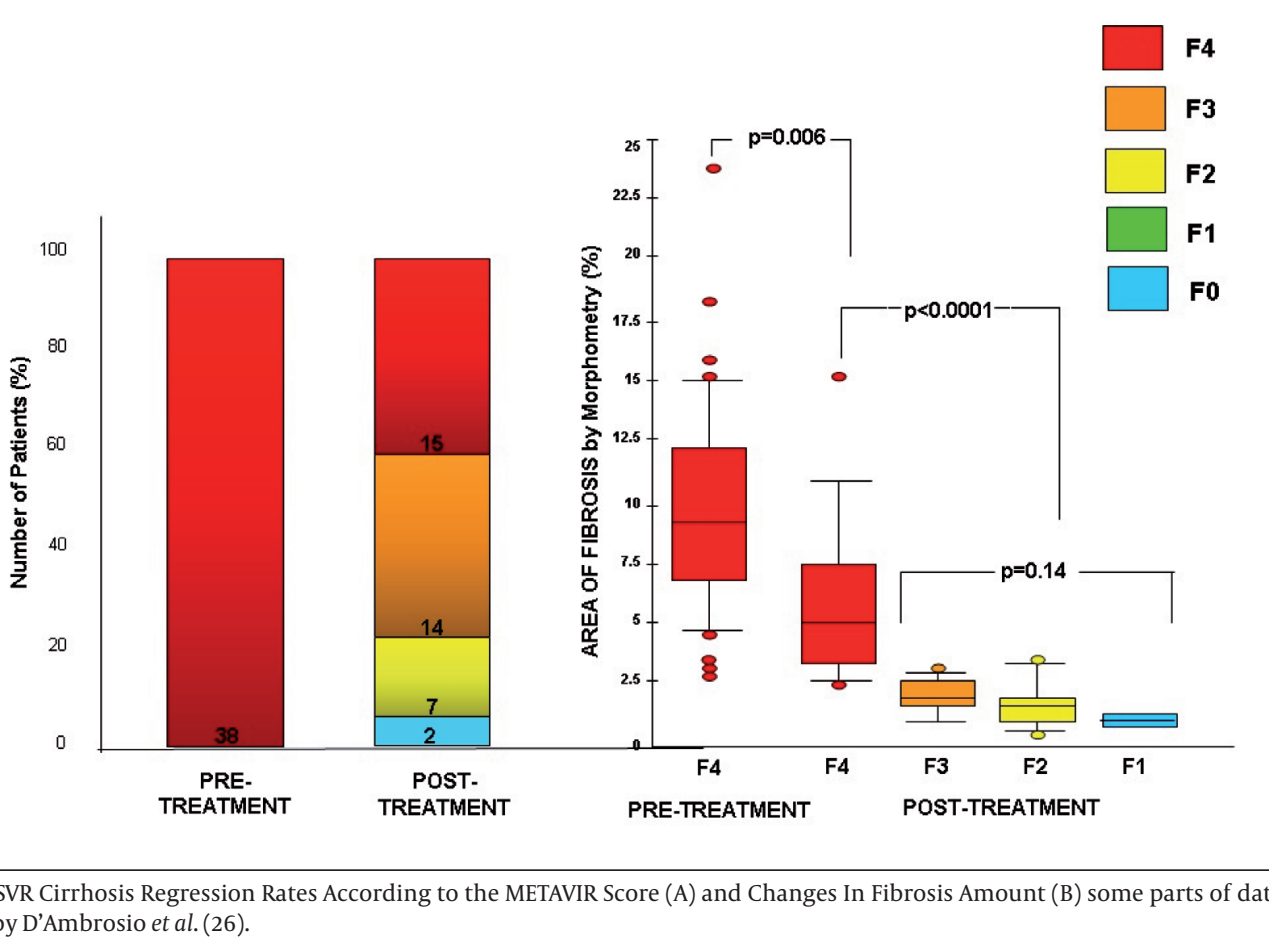
Post-SVR Cirrhosis Regression Rates According to the MeTAVIR Score (A) and Changes In Fibrosis Amount (B) some parts of data are derived from the Study by D’Ambrosio et al. [26].

The reasons why persistent viral eradication in HCV cirrhotics is able to reduce rates of liver-related complications may be due to the abolition of carcinogenetic action on the HCV, in the case of HCC development, as well as to the restoration of a pre-cirrhotic liver architecture that has been shown to occur following a SVR [11][27][28][29][30][31]. Indeed, several studies have demonstrated fibrosis regression occurring after the achievement of an SVR in HCV patients with any degree of liver damage prior to the start of antiviral therapy [11][27][28][29][30][31][32][33][34][35] (Table 2). However, only a few studies have focused on cirrhotic patients. Moreover, the clinical meaningfulness of fibrosis/cirrhosis regression has not been fully investigated yet, currently it is still unclear whether the reduction in the incidence of liver-related complications in cirrhotic patients with a SVR actually relies on a histological improvement and, in particular, on the occurrence of cirrhosis regression. Many studies have provided histological data obtained after the achievement of a SVR in cirrhotics, although in most cases the histological outcome was not among the primary endpoints of that study. The reported rates of cirrhosis regression range between 24% and 100%, with 10 studies reporting any degree of cirrhosis regression [11][32][33][34][35]. Unfortunately, these studies are hardly comparable, due to methodological issues such as differences in the definition of cirrhosis regression and discrepancies in the semi-quantitative scores used, as well as differences in terms of the post-treatment follow-up duration. Moreover, despite the fact that many studies enrolled large cohorts of patients with chronic HCV infection, the prevalence of pre-treatment histological diagnosis of cirrhosis was relatively low, thus imposing caution when interpreting the prevalence of cirrhosis regression. Poynard et al. [28] were among the first to investigate retrospectively the impact of a SVR on liver histology, by analyzing data from four randomized therapy trials of patients with pre- and post-treatment liver biopsies. Despite a short post-treatment follow-up period (i.e. mean interval between pre- and post-treatment liver biopsies ≤ 21 months), they found that cirrhosis regression occurred in 68% of patients who achieved a SVR. The benefit of persistent viral eradication on liver fibrosis was subsequently demonstrated in another French study (26, in which the rates of cirrhosis regression were significantly higher among patients with a SVR (24% vs. 2%, P = 0.02). The difference in the rates of cirrhosis regression reported in these studies might be explained by some important methodological issues, such as the limited number of cirrhotics included in the analysis and the timing of the post-treatment follow-up liver biopsy. More recently, two European studies [11][31] have investigated the relationship existing between the achievement of a SVR and the incidence of cirrhosis regression following interferon and ribavirin (IFN/RBV) combination treatments. Noteworthy, in both of these studies, cirrhosis was defined according to the METAVIR score (F4), even if cirrhosis regression was defined as a reduction of 2 and 1 point, respectively. Overall, cirrhosis regression was demonstrated in 44% and 61% of the patients, respectively, with this slight difference probably being related to the different definition of the event. In the Italian-French collaborative study [31] conducted on 38 HCV cirrhotics treated with IFN-based regimens, an improvement in the liver architecture as assessed by the METAVIR score was demonstrated in more than half (61%) of the patients, after a median follow-up of 61 months following the achievement of a SVR. Interestingly, in this study a reduction in the amount of fibrosis as assessed by morphometry was demonstrated in the near totality of the patients, even in the absence of cirrhosis regression (Figure 1). The study conducted by Mallet et al. Not only demonstrates high rates of cirrhosis regression after a shorter period of follow-up following a SVR (median 11 months), but also has the added benefit of providing information on a further follow-up period, post the second liver biopsy. This allowed identification of a difference in the incidence of liver-related clinical events among patients who regressed or those who did not following a SVR. Indeed, while patients still staged F4 after a SVR developed some clinical events (i.e. 3 liver-related deaths/OLT, 3 HCC and 1 variceal bleeding), none of the patients with cirrhosis regression showed any liver complications. This French study de facto suggests that the occurrence of liver-related events post-SVR relies on architectural improvement more than on HCV clearance. These data results obviously need external validation before they can be translated confidentially into clinical practice, but they may help in improving the clinical follow-up of HCV cirrhotics with a SVR. Indeed, post-treatment surveillance for HCC development and/or variceal development/enlargement could be suspended in patients with a histological improvement, hence reducing health care costs in the management of SVR cirrhotics.

**Table 2:** Rates of Cirrhosis Regression Among SVR and Non-SVR Patients, According to the Different Semi-Quantitative Staging Scores Used

	**Patients, No.**	**Cirrhotics, No.**	**Non-SVR Cirrhotics, No.**	**SVR Cirrhotics**	**Follow-up, mo^a^**	**Staging Scoring System**	**Cirrhosis Regression Rates Among non-SVR, No. (%)**	**Cirrhosis Regression Rates Among SVR , No. (%)**	***P value***
Reichard et al. (1999) [32]	23	4	0	3	na (25-302)	Scheuer	na	3 (100)	na
Shiratori et al. (2000) [27]	593	62	30	24	44 (12-120)	METAVIR	9 (30)	11 (46)	0.27
Poynard et al. (2002) [28]	3010	153	116	37	< 24	METAVIR	50 (43)	25 (68)	0.01
Arif et al. (2003) [34]	56	15	9	6	na (0-66)	Ishak	4 (44)	5 (83)	0.29
Pol et al. (2004) [29]	64	64	47	17	na	METAVIR^c^	1 (2)	4 (24)	0.02
Mallet et al. (2008) [11]	96	96	57	39	11 (na)	METAVIR^c^	1 (2)	17 (44)	< 0.0001
Everson et al. (2008) [35]	184 b	140	144^b^	40^b^	0	METAVIR	34 (24)^b^	20 (50) b	0.003
George et al. (2009) [30]	49	8	0	8	56 (42-72)	Ishak	na	6 (75)	na
D’Ambrosio et al. (2012) [31]	38	38	0	38	61 (48-104)	METAVIR	na	23 (61)	na

^a^ Median (range) from SVR achievement (i.e. 6 months after end of treatment)

^b^ In the study by Everson et al, F3 and F4 patients were analyzed cumulatively, and clinical and histological data for F4 patients (n=140), only, are missing.

^c^ Cirrhosis regression evaluated as a reduction in at least 2 points

## 4. Peginterferon and Ribavirin Efficacy Among Patients With HCV Cirrhosis

Although the achievement of a SVR in HCV cirrhotics does not completely abrogate the risk of liver-related complications, SVR is still clearly a sine qua non condition for the achievement of clinical benefits following IFNbased treatments. Unfortunately, cirrhosis is associated with reduced rates of SVR both in HCV-1 and HCV-4 patients as well as in HCV-2 and HCV-3 patients [36]. In HCV-1 and HCV-4 patients, cirrhosis is usually associated with reduced rates of on-treatment response, while in the more Pegylated interferon (PegIFN)/RBV sensible HCV2 and HCV-3 genotypes the main reason for treatment failure is an increased risk of post-treatment relapses [37]. The exact reason why cirrhosis determines this genotype-dependent pattern of treatment failure, as well as the mechanisms through which it reduces the antiviral effect of PegIFN / RBV is still unknown. However, it is probable that they might rely on the anatomical subversion of the liver, which might prevent optimal interactions between IFN and target liver cells. A recent study [38] demonstrating PegIFNalpha-2a to be less negatively influenced in terms of SVR rates, by the degree of fibrosis/cirrhosis compared to PegIFNalpha2b, indirectly supports this concept. PegIFNalpha-2a is in fact characterized by a small volume of distribution making it more likely that the drug concentrates directly into the liver, a PK property [37] that might bypass the negative impact of advanced fibrosis/cirrhosis on the SVR rates. Unfortunately, to date, no attempts to improve SVR rates in cirrhotics by manipulating the standard of care PegIFN/RBV regimen in terms of doses or duration of treatment have provided any added clinical benefits, suggesting that patients with cirrhosis should be treated as patients without cirrhosis and receive the standard regimen care in terms of dose and duration.

## 5. Safety and Tolerability of PegIFN/RBV in HCV Related Cirrhotic Patients

The standard combination of PegIFN and RBV is associated with many adverse events, including flu-like syndrome (28%; range 17%-67%), depression (23%; range 15%34%), fatigue (55%; range 42%-66%), and haematological abnormalities (15%; range 6%-17%). However, the safety and tolerability among patients with compensated cirrhosis undergoing this treatment does not differ from those in non-cirrhotic patients [40][51]. Similarly, discontinuation rates in compensated cirrhotics are similar to those observed among non-cirrhotic patients [40][42][43][44][52][53][54], even if patients with more advanced liver disease are more likely to require dose reduction [36][44][53], particularly as a result of haematological side effects [52][54][55]. Importantly, clinical decompensation rates in cirrhotic patients undergoing IFN-based regimens are negligible (0-3%) [52][53][55], maybe as a consequence of careful patient selection, excluding those with more advanced liver disease, as these patients still remain at increased risk of liver function deterioration. Indeed, mainly due to the increased risk of clinically relevant anaemia, thrombocytopenia and neutropenia predisposing to the occurrence of bacterial infections and consequently impairment of liver function, the treatment of decompensated cirrhotics should be avoided and considered only if patients are included on a liver transplantation list. Therefore antiviral treatment of Child-Pugh score A cirrhotic patients should be encouraged, whereas it is absolutely contraindicated in Child C patients; further prospective studies are required in order to understand whether IFN/RBV treatment is safe and effective in Child B cirrhotics.

## 6. PegIFN Maintenance Therapy to Improve the Outcome of HCV Compensated Cirrhosis

Since the vast majority of HCV cirrhotics fail to achieve a SVR to IFN / RBV therapy, they therefore remain at high risk of HCC, liver decompensation and variceal bleeding [7][8][9][10][11][19], alternative treatment regimens have been explored during the last decade. The one that has probably had more scientific and commercial support is the administration of a long course of low dose interferon, the so called maintenance therapy that was advocated at the end of the 1990’s. The scientific and clinical rationale being several retrospective studies [20][54] carried out in the late 1990’s showing that IFN therapy was associated with a reduction in the rates of HCC development as compared to clinical observation, as well as the well-known anti-proliferative effects of IFN on various cell lines. Three randomized controlled studies [56][57][58] were designed to assess if a long term course of low dose PegIFN therapy could reduce the rate of liver-related complications in patients with advanced fibrosis. Direct comparison of the studies’ results is partially precluded by differences in the patients’ characteristics and in the assigned treatment regimens; however, they unanimously failed to demonstrate any positive impact of PegIFN maintenance therapy on survival as well as on the incidence of HCC rates. The same also holds true for the extended follow-up analysis of the HALT-C trial [59], where no benefit was seen in the overall population, but a small benefit in terms of HCC reduction in patients receiving maintenance therapy was seen in patients classified as cirrhotics at baseline compared to those with advanced fibrosis (cumulative HCC incidence: 6.8% vs. 15.5%, P = 0.01). Thus the issue of HCC prevention in IFN nonresponders remains unresolved. The only beneficial effect seen in two studies [57][58] was a reduced rate of EV development or variceal bleeding in patients receiving PegIFN maintenance therapy compared to the control group, that could suggest an eventual role for PegIFN in the prevention of portal hypertension complications more than in the develop ment of HCC. Still, given the unpleasant side effect profile of PegIFN maintenance therapy, coupled with only marginal clinical benefits, long term low dose PegIFN therapy should not be given to cirrhotic patients outside of controlled clinical trials.

## 7. Conclusions

**Figure 2:**
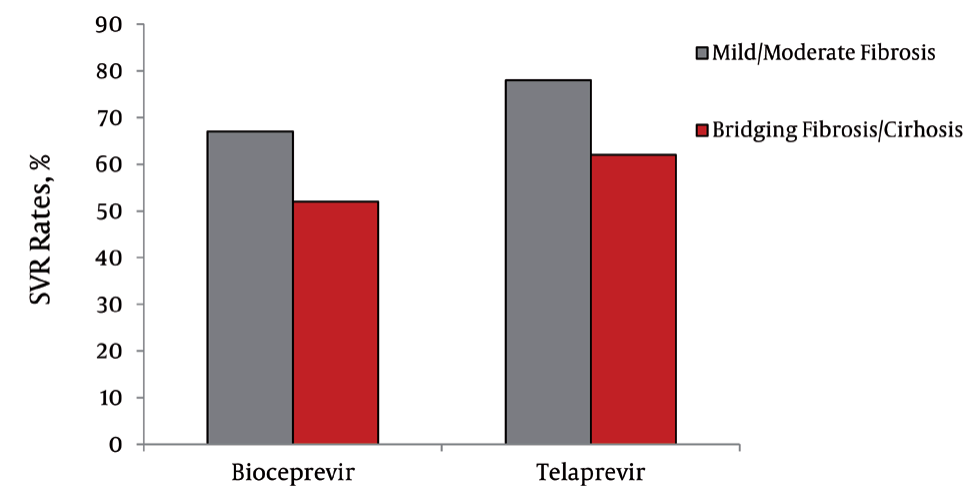
Sustained Virological Response Rates in Telaprevir and Boceprevir Phase III Studies on Naïve Patients, Stratified by Degree of Liver Fibrosis

More than 20 years after the discovery of HCV, clinicians are able to cure almost 50% of their patients with IFN-based therapies. Unfortunately, one of the highest need groups, those with cirrhosis, still attains subpar SVR rates, due to the reduced efficacy of the IFN molecule on a liver with such significant anatomical changes. The introduction of the first class of directly acting antivirals (DAAs) that will become commercially available in most countries worldwide in 2012, will only partially solve this problem. Indeed both of the NS3 protease inhibitors, telaprevir and boceprevir, need to be administered in combination with PegIFN and RBV, and require former standard of care therapy to elicit some form of anti-HCV activity to maximise SVR rates [60]. For this reason, patients with cirrhosis will still achieve lower SVR rates with triple therapy regimens compared to patients without cirrhosis (Figure 2) [61][62]. This is even more relevant in patients with a previous treatment failure to PegIFN plus RBV, where in the presence of cirrhosis, disappointing SVR rates to a telaprevir based regimen have been reported by Phase III trials [63][64] (Figure 2). It is probable therefore, that to finally bypass the negative role of cirrhosis as a moderator of treatment failure, we will need to develop therapeutic strategies that do not rely on the broad antiviral effect of IFN [65]. In the meantime, treating patients with HCV related cirrhosis will continue to remain a challenge, both for clinicians as well as for our patients, a challenge that, however, can reward both immensely if success is achieved.
